# A Hybrid Recommendation Approach for Viral Food Based on Online Reviews

**DOI:** 10.3390/foods10081801

**Published:** 2021-08-04

**Authors:** Cen Song, Qing Yu, Esther Jose, Jun Zhuang, He Geng

**Affiliations:** 1 School of Economics and Management, China University of Petroleum, Beijing 102249, China; songcen22@126.com (C.S.); 13121188206@163.com (Q.Y.); 2 Department of Industrial and Systems Engineering, University at Buffalo, Buffalo, NY 14260, USA; estherjo@buffalo.edu; 3 Kunlun Trust Co., Ltd., Beijing 100033, China; genghe8888@163.com

**Keywords:** viral food, sentiment analysis, hybrid recommendation approach, text analysis

## Abstract

Nowadays, there are many types of viral foods and consumers expect to be able to quickly find foods that meet their own tastes. Traditional recommendation systems make recommendations based on the popularity of viral foods or user ratings. However, because of the different sentimental levels of users, deviations occur and it is difficult to meet the user’s specific needs. Based on the characteristics of viral food, this paper constructs a hybrid recommendation approach based on viral food reviews and label attribute data. A user-based recommendation approach is combined with a content-based recommendation approach in a weighted combination. Compared with the traditional recommendation approaches, it is found that the hybrid recommendation approach performs more accurately in identifying the sentiments of user evaluations, and takes into account the similarities between users and foods. We can conclude that the proposed hybrid recommendation approach combined with the sentimental value of food reviews provides novel insights into improving the existing recommendation system used by e-commerce platforms.

## 1. Introduction and literature Review

Since Industrial 4.0 has profoundly changed the dynamics of the whole industry in the face of rapid and continuous development, viral foods can be allowed to flourish (Akyazi et al. [1]). Viral foods refer to foods that are widely welcomed by the public for a period of time through online marketing and publicity. In recent years, the types of viral foods have been increasing, and an increasing number of young people are keen to try a variety of viral foods, which have become a major component of young people’s consumption. The packaging and flavors of viral foods are becoming increasingly novel, and their types are more diverse. Online review-driven viral food has become more popular recently during the COVID-19 pandemic; Cranfield [2] analyzes income and time constraints as well as price effects that impact the demand for food across sociodemographic characteristics. The COVID-19 crisis is an opportunity and a challenge for viral foods. With unique taste, viral foods promote high consumption and economic growth in the food industry. However, with the rapid development, safety, standards, quality, and other issues have become the concern of regulatory authorities and public opinions (Galanakis et al. [3]).

It is the common desire of consumers to quickly and accurately find viral foods that meet their own tastes among a wide range of online platform recommendations, and save unnecessary search time and waste of funds. The viral food recommendation systems on different platforms not only actively connect users with information, but also filter through available information to provide users with valuable and relevant information. Various recommendation approaches can achieve precision marketing and provide exclusive food recommendations for consumers. The traditional food recommendation system only provides recommendations for fixed popular viral foods, which narrows the choices of consumers and cannot meet the preferences of all consumers. Thus, the user’s satisfaction is lower with the fixed recommendation system. Hence, personalized recommendation is required by major e-commerce platforms, which use an appropriate recommendation approach to connect personalized foods to users with different preferences. These approaches mainly rely on the user’s historical behavior information. The collected data are analyzed and used to improve the click-through rate and order rate. Therefore, the ingenious application of the personalized recommendation approach to the food recommendation system is beneficial in improving the accuracy of the recommendation.

### 1.1. Research on Recommendation Approaches

The main recommendation approaches include content-based recommendation, collaborative filtering recommendation, and the hybrid recommendation approach, among which the collaborative filtering algorithm has the most extensive application thanks to its simple principle and high practicability [4]. However, in application, the scoring matrix is easily affected by the quality of the rating as well as the new users and new products. In order to make full use of all the scoring information, Anand and Bharadwaj [5] used global and local similarity calculation methods, and selected the nearest neighbors by combining them.

In the aspect of similarity threshold calculation, based on an effective threshold-based neighbor selection in collaborative filtering, Kim and Yang [6] used the substitute neighbors of the test customer to find the proper neighbors and obtain a good prediction quality. Different matrix reduction and filtering methods have been proposed in the current research field. Wang et al. [7] developed a user-based and project-based fusion collaborative filtering algorithm theory, which effectively alleviated the problem of data sparsity and improved the accuracy of near-neighbor search. Sharif et al. [8] used matrix factorization and implemented singular value decomposition (SVD) to develop a recommendation approach that is superior to other algorithms of collaborative filtering in dealing with the sparsity data problem. Alhijaw and Kilani [9] presented a novel genetic-based recommender system to alleviate the cold start and sparsity problems by considering the individual in the population as a potential recommendation list, which achieves more accurate predictions than the alternative methods regardless of the number of neighbors, K.

The hybrid recommendation approach combines the advantages of different algorithms and is widely used. Thorat et al. [10] provided an overview of recommended systems that include the collaborative filtering, content-based filtering, and hybrid approaches to a recommendation system, which can be applied in many fields such as e-commerce sites, businesses, jokes, restaurants, financial services, life insurance, and Twitter. Pal et al. [11] proposed an improved hybrid content-based collaborative filtering algorithm for movie recommendations, which performs better than pure collaborative filtering and singular value decomposition. Yang et al. [12] constructed a hybrid job recommendation system by applying statistical relational learning to determine the combination weight of content recommendation and collaborative filtering recommendation results, allowing for a tuning of the trade-off between the precision and recall of the system in a principled way. Combining a content-based recommendation method, a collaborative filtering recommendation method, and a complementarity-based recommendation method, Li et al. [13] proposed a novel hybrid system to recommend Q&A documents to alleviate overload based on the Fermat point. Yue et al. [14] combined the project- and user-based recommendation methods to generate a recommendation, and used the similarity weight calculation method to fill the vacancy value by combining the average value and the calculated value.

Owing to the accessibility of commodity evaluations and labels, the hybrid recommendation method (content recommendation and collaborative filtering recommendation) has become an important direction for most recommendation systems. To the best of our knowledge, past works have not applied the collaborative filtering algorithm in the field of viral food. This paper fills the research gap by constructing a hybrid recommendation approach based on the user-based and content-based recommendation approaches to recommend viral food to users.

In summary, collaborative filtering and a hybrid algorithm based on content recommendation are often used in current recommendation approach research, and their recommendation results are obviously better than the recommendation results of a single algorithm. This paper takes several typical viral foods as the research objects, and uses the label similarity of viral food content and online review scores to obtain the recommendation prediction results.

### 1.2. Sentiment Analysis Research

Text sentiment analysis refers to the use of machines to automatically read, select, process, summarize, and predict the sentimental attitude and polarity of text. Medhat et al. [15] identified that textual sentiment analysis techniques typically use a machine learning approach, a lexicon-based approach, or a hybrid approach.

The machine learning method has high accuracy, but it is mostly used for classification and has the disadvantage of over-reliance on manual annotation. Sentiment dictionary analysis matches words having sentiment characteristics in the text with words in the sentiment dictionary, and calculates the sentiment category of the whole sentence according to the sentiment polarity assigned by the dictionary. By assigning appropriate sentimental values to sentimental words, not only can the positive and negative sentimental tendencies of the text be obtained, but also the specific sentimental value of the text can be calculated [16]. Al-Natour and Turetken [17] found that sentiment analysis is very effective in capturing the true sentiment of a review and can be used as a complement or an alternative to star ratings. Different sentiment analysis tools are examined and found to have varying accuracies; in general, however, sentiment analysis accurately captures the tone of the reviews.

At present, there are many studies that use sentiment analysis technology in the recommendation field. Alaei et al. [18] applied different approaches to the sentiment analysis of tourism reviews, including machine learning, dictionary-based, semantic, and hybrid approaches. Song et al. [19] analyzed and mined users’ sentiments and opinions using natural language processing, after which latent Dirichlet allocation (LDA) and k-means were used to extract and cluster topics from the posts on take-away food safety. Chintalapudi et al. [20] implemented text mining in seafarers’ medical documents and performed sentimental analysis by adopting both lexicon and naïve Bayes’ algorithms to generate knowledge of medical issues.

The content-based recommendation approach can make personalized recommendations to users and overcome the problems of cooling startup and matrix sparseness. However, it is difficult to explore the potential interests of users and obtain the preference characteristics of new users. The user-based collaborative filtering recommendation approach can discover the user’s potential interests and recommend new information to the user. The advantages and disadvantages of the two algorithms are complementary. Therefore, the hybrid recommendation approach is proposed, taking into consideration the characteristics of food review and label attribute data, which combine the user-based recommendation approach with the content-based recommendation approach. It uses Chinese language processing and sentiment analysis technology to quantify the sentiment values of the viral food evaluations. This approach specifically recommends viral food to users, and provides insight into ways to improve the recommendation systems of e-commerce platforms.

The rest of the paper is organized as follows. Section 2 proposes a hybrid recommendation model based on user evaluation sentiment analysis, where a custom sentimental dictionary is constructed and the sentiment values of user evaluations are calculated. Section 3 compares the hybrid recommendation approach with other approaches, and conducts an empirical study. Section 4 summarizes the work and discusses its limitations and future directions.

## 2. The Proposed Hybrid Recommendation Approach

### 2.1. The Technical Aspect of the Hybrid Recommendation Approach

Through the collection and analysis of data from the JD.com (accessed on 9 May 2021) platform and existing recommendation approaches, this paper calculates the similarity between the sentiment value of users’ reviews and the food attribute label to reduce the error of food recommendation. The approach in this paper is composed of two parts. First, the sentiment value of user reviews is calculated based on the constructed sentiment dictionary. Then, the hybrid recommendation technique is applied.

HowNet is a very good semantic resource or linguistic knowledge base in China. It has a fine semantic description system with more than 2000 sememes, and annotates the meanings of the concepts represented by more than 100,000 Chinese and English words. Using the HowNet sentiment dictionary and the simplified Chinese sentiment polarity dictionary as the basic sentiment dictionaries, the Semantic Orientation Pointwise Mutual Information (SO-PMI) algorithm is used to extract a viral food domain dictionary from user reviews. After deduplication filtering, a custom dictionary for sentiment analysis is constructed.

**Figure 1:**
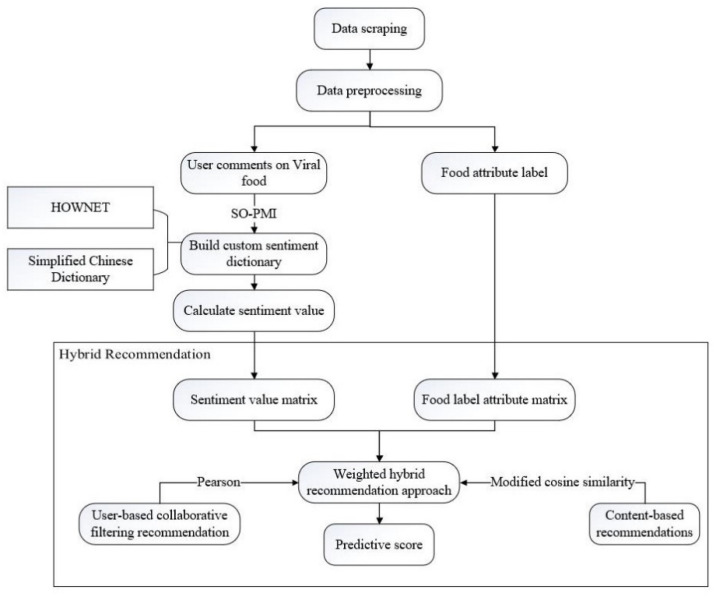
The technical route of the hybrid recommendation approach.

To apply the hybrid recommendation technique, the sentiment values are used to form the sentiment value scoring matrix. The food types and additional labels provided by the JD.com (accessed on 9 May 2021) platform are combined to create the food attribute labels. The modified cosine similarity is used to calculate the similarity between the food and the user. Based on the sentiment value scoring matrix, the similarity among users is calculated using the Pearson similarity method. Then, the top N-type foods with the highest similarity are predicted according to the similarity ranking. The technical route of the hybrid recommendation approach is shown in Figure 1.

The proposed hybrid recommendation approach is Hybird = w*CB + (1 − w)*UserCF, where Hybird is the proposed hybrid recommendation approach, w is the weight coefficient, CB is the content-based recommendation approach, and UserCF is the user-based collaborative filtering recommendation approach.

### 2.2. Sentiment Analysis of Online Evaluations

#### 2.2.1. Construction of a Custom Sentimental Dictionary Based on SO-PMI

This paper constructs a dictionary in the field of food based on the SO-PMI algorithm. The SO-PMI algorithm uses seed words as the basis for construction. If the probability of a certain word and the seed word appearing at the same time is large, the word has the same sentimental tendency as the seed word. Therefore, it is necessary to identify the seed words that appear in user reviews with high frequencies, as well as their sentimental tendencies.

**Figure 2:**
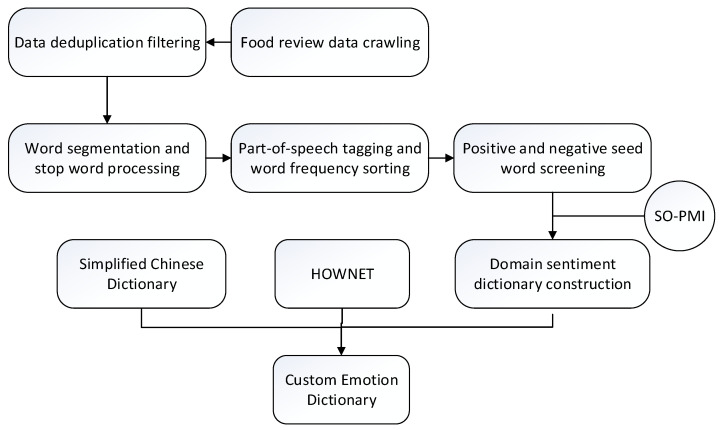
The construction process of a custom sentimental dictionary.

The detailed description of the construction process shown in Figure 2 is as follows:

Step 1: Crawl a variety of food reviews on JD.com (accessed on 9 May 2021), and de-duplicate and filter the crawled data according to the requirements, leaving real and effective data.

Step 2: Read the review data after deduplication and screening, one by one. Use Jieba technology for word segmentation, and then delete the words in the stop vocabulary list after the word segmentation.

Step 3: Perform part-of-speech tagging on the words after the stop words are removed, then sort the top 3000 according to the word frequency and select the positive and negative sentimental words related to the food field to form a seed set.

Step 4: Use the SO-PMI algorithm to calculate the SO-PMI correlation values of other words in the reviews with the seed words. Sort according to the SO-PMI values, and manually delete words that are not sentimentally inclined and have low SO-PMI correlation values. Construct a domain-specific sentimental dictionary.

Step 5: Combine the HowNet sentiment dictionary, the simplified Chinese sentiment polarity dictionary, and the domain-specific sentiment dictionary. Then, perform deduplication and screening to construct a new custom domain-specific sentiment dictionary.

#### 2.2.2. Sentiment Value Calculation Based on Custom Dictionary

**Figure 3:**
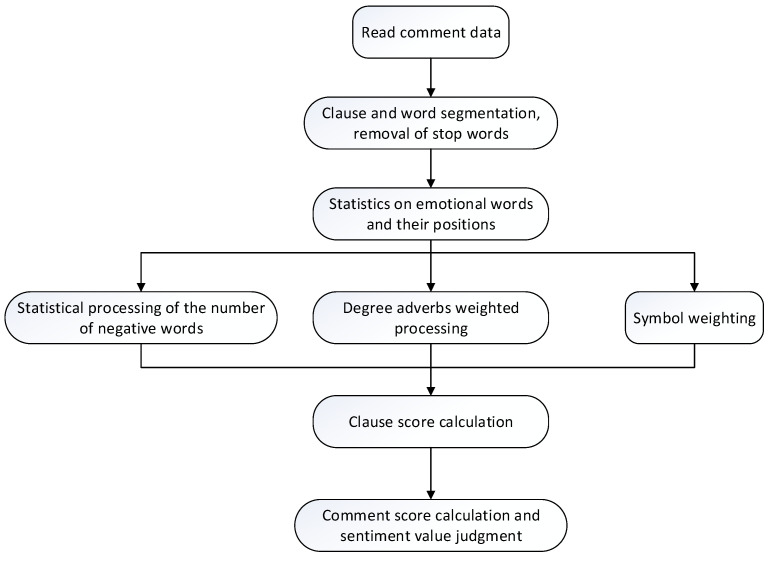
The calculation process of a sentiment value.

The sentiment analysis based on the sentiment dictionary is realized by matching and summing the Corpus words with the words in the custom sentiment dictionary. First, determine the polarity of the words in the review text after individual word segmentation. If it is a positive word, the number of positive words is increased by 1. If it is a negative word, the number of negative words is increased by 1. At the same time, it is necessary to judge whether there is a degree adverb before the sentimental word. It is necessary to assign different weights to different types of degree adverbs and add them to the number of sentimental words. Similarly, if “!” or “?” symbols exist at the end of the reviews, the sentiment values are higher. The sentimental tendency of the review text is determined using the sentiment value of the whole sentence. If the score is greater or less than zero, the sentimental tendency is positive or negative, respectively. A score of zero indicates a neutral sentimental tendency. The calculation process of a sentiment value is shown in Figure 3.

#### 2.2.3. Accuracy Evaluation Index for the Sentiment Analysis

The accuracy of the sentiment analysis results is determined by the precision rate, recall rate, and F1 value, as calculated in Formulas (1)–(3), respectively.

(1) $$\mathrm{Precision}(P)=\frac{TP}{TP+FP}$$

(2) $$\mathrm{Recall}(R)=\frac{TP}{TP+FN}$$

(3) $$F1=\frac{2*P*R}{P+R}$$

Here, *FP* represents the number of false positive reviews in the test set, *TP* represents the number of true positive reviews in the test set, *TN* represents the number of true negative reviews in the test set, and *FN* represents the number of false negative reviews in the test set.

### 2.3. Hybrid Recommendation Approach Based on Sentiment Analysis

**Figure 4:**
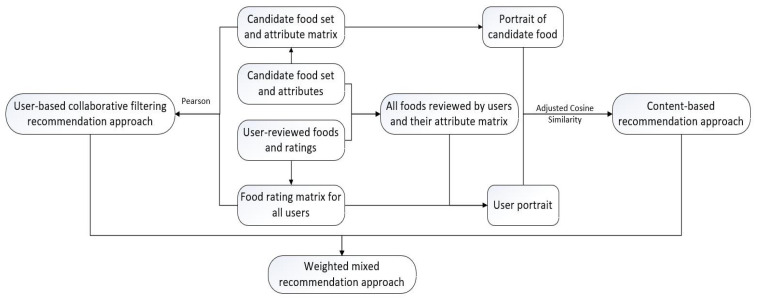
Flow chart depicting the hybrid recommendation approach.

Figure 4 is a flow chart depicting the process of the hybrid recommendation approach.

The detailed description of the flow chart is as follows:

Step 1: Manually segment and filter the 50 crawled food names to obtain the attribute information corresponding to each food, and then convert each food name and attribute information into a candidate food set and attribute binary matrix.

Step 2: Combine all the foods evaluated by all users and their corresponding sentimental scores into a user’s food score matrix.

Step 3: After combining the food set and attribute data with the food and scoring data evaluated by the user, transform them into a matrix of all the foods evaluated by the user and their binary attributes: 0 and 1.

Step 4: From the user food scoring matrix, all the foods evaluated by the user, and their binary attribute matrix, the user portrait can be obtained. Use the modified cosine similarity calculation method to calculate the similarity between the candidate food portrait and the user portrait, sort the resulting food recommendation set, and finally choose the top N recommended foods to complete the content-based recommendation approach.

Step 5: Let K be the number of nearest neighbors selected. Based on the user’s food scoring matrix, use the Pearson similarity technique to calculate similarity with the K nearest neighbors or users, and sort the similarity in descending order. According to the preference of the nearest neighbors, the recommended food is selected from the array of foods, and the user-based collaborative filtering recommendation approach is completed.

Step 6: Assign different weights to the content-based recommendation and the user-based collaborative filtering recommendation so that the weights sum up to 1. Then, the top N recommended foods make up the output, completing the hybrid recommendation approach.

The accuracy of the recommended food affects the performance of the recommendation approach. Therefore, the mean absolute error (MAE) is used as the measurement index to judge the accuracy of the recommendation result. MAE is the average value of the absolute difference between the predicted result and the actual result. The smaller the MAE, the better the accuracy of the prediction approach. MAE is calculated as shown in Formula (4).

(4) $$\mathrm{MAE}=\frac{\sum_{i=1}^{n}\left|f_{i}-y_{i}\right|}{n}$$

where $f_{i}$ represents the predicted value and $y_{i}$ represents the true value.

## 3. Recommendation Approach Application and Comparative Analysis

### 3.1. Data Collection and Preprocessing

**Figure 5:**
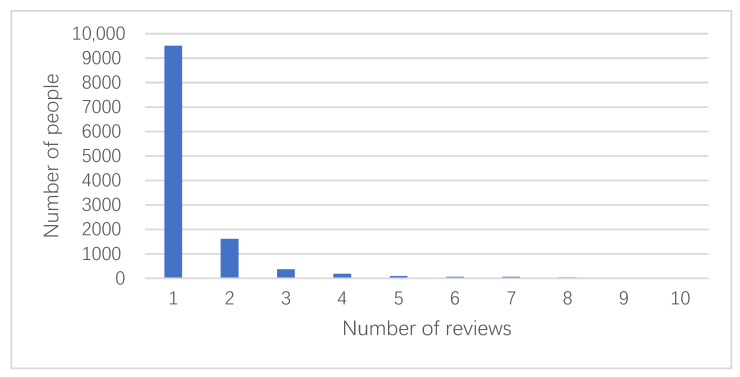
Statistics of the number of user reviews.

The main purpose of this paper is to analyze users’ product reviews and product attribute data from JD.com (accessed on 9 May 2021), where the Octopus collector is used to crawl the food attributes of 50 products and 23,680 reviews. The crawled data include five data items: product name, user name, review content, review date, and star rating. The statistical results are shown in Figure 5. The number of people who reviewed 1–2 times form the majority, with their contribution to the recommendation approach having less significance. Therefore, the data are randomly deleted. Additionally, the scalping data of reviews of merchants are also deleted, which results in 12,577 pieces of data for approach construction.

### 3.2. The Application of the Hybrid Recommendation Approach

#### 3.2.1. Sentiment Analysis Based on Users’ Reviews

Sentiment value calculation: With the food review dataset, based on the SO-PMI method, a self-defined domain-specific dictionary is constructed and sentiment values are calculated. After word segmentation, the words are counted and sorted, where the words with sentiment and related to the food field are manually selected from the top 3000. The 50 positive words and 50 negative words are selected as the seed set. For example, some frequent positive words include enough, exquisite, value for money, guarantee, new, high quality and inexpensive, trust, and so on. Some frequent negative words include unnecessary, awkward, give up, disadvantageous, resist, endless, any carelessness, spit out, and so on. Then, using the seed words, the SO-PMI algorithm is used to construct the positive sentiment dictionary and the negative sentiment dictionary. The words without sentimental tendency are manually deleted to complete the construction of the custom sentiment dictionary.

The experiment normalizes the sentiment value output and sets the upper and lower limits. The sentiment value of a review with a sentiment value greater than 10 is defaulted to 10, and a sentiment value less than −10 is defaulted to −10.

Comparison of sentiment analysis results: According to the judgement of the positive and negative values, reviews are divided into three categories: positive, neutral, and negative. When the sentiment value is greater than 0, the review is positive. When the sentiment value is less than 0, the review is negative. When the sentiment value is equal to 0, the review is neutral. The test set consisting of 500 reviews is manually labeled and compared with the machine classification results. The evaluated accuracies of the sentiment analyses based on the HowNet sentiment dictionary, simplified Chinese sentiment dictionary, and custom sentiment dictionary are shown separately in Table 1.

Owing to the improved sentiment vocabulary, it can be seen that the custom sentiment dictionary has higher precision and is better, overall, at judging the sentiment tendency of reviews. The custom sentiment dictionary is used in the remainder of the paper as it has the highest precision, recall, and F1 score.

#### 3.2.2. Analysis of Hybrid Approach Results

Owing to the imbalance of user evaluation scores on the JD.com (accessed on 9 May 2021) platform, the number of 5-star evaluations accounted for more than 95% of the total number of evaluations, rendering the user evaluation scores ineffective. Therefore, this paper uses the sentiment score obtained in the last section as the user’s evaluation score. The data are divided such that the training set is 70% of the total data set and the test set is 30% of the data set.

**Figure 6:**
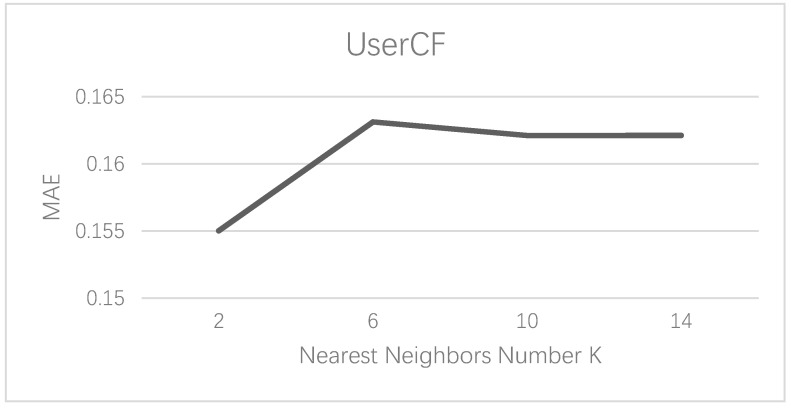
The influence trend of K on mean absolute error (MAE).

As the results of the user-based collaborative filtering (CF) recommendation approach are affected by the number of nearest neighbors, K, its optimal value is selected by comparing the MAE values for different levels of K. Figure 6 shows the influence of the value of K on the MAE value. As K increases, the MAE value has an upward trend. When K is equal to 2, the MAE is the lowest, which indicates that the prediction result is the most accurate.

**Figure 7:**
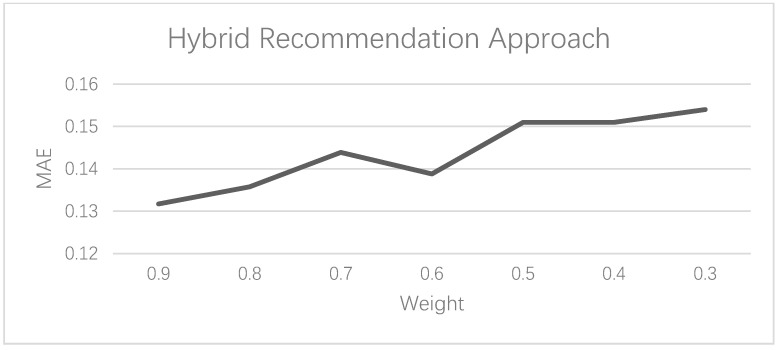
MAE values of the hybrid recommendation approach under different weights.

Given that the optimal value of K is 2, the parameters of the user-based collaborative filtering recommendation approach are determined. The hybrid recommendation approach can generate different recommendation results given different weights. Figure 7 shows the different MAE values of the hybrid recommendation approach at different weights. The weight, W, represents the weight assigned to the content-based recommendation approach (CB), and the weight assigned to the user-based collaborative filtering recommendation approach (CF) is 1 − W. It can be seen that, as the value of W increases, the value of MAE has an obvious upward trend. Therefore, when the weight is 0.9, the recommendation of the hybrid approach is the best.

### 3.3. Comparative Analysis of the Recommended Approaches

By comparing the recommendation results of the content-based recommendation approach with an optimal K, the user-based collaborative filtering recommendation approach, and the hybrid recommendation approach with the optimal weight, the approach with the best performance can be identified.

Table 2 shows MAE values corresponding to the three recommendation approaches. The MAE value of the content-based recommendation is 0.131712 and the MAE value of the hybrid recommendation is 0.131704 when the weight, W, is 0.9. Both of these values are lower than the MAE value of the user-based collaborative filtering recommendation, which is 0.155015 when K is 2. The recommendation result of the hybrid approach is slightly better than the recommendation result of the user-based collaborative filtering.

However, there is no obvious improvement achieved by using the hybrid recommendation approach over the content-based recommendation approach. Additionally, Table 3 shows the precision, recall, and F1 values of the three algorithms. The user-based recommendation approach has the lowest precision. The hybrid recommendation approach and the content-based approach do not differ significantly in precision and the F1 score. In recall, however, the hybrid approach performs a little better than the content-based approach.

Owing to the small amount of data, the improvement of the proposed hybrid model is relatively insignificant, and the superiority of the hybrid approach is not firmly established. The hybrid recommendation approach considers the influences of both user similarity and food attribute similarity, which makes it a more comprehensive approach.

### 3.4. Empirical Study

An empirical study is conducted to validate the hybrid recommendation approach based on text analysis as proposed in this paper, where the time period covered by the empirical analysis is June 2015 to April 2021. The hybrid recommendation approach is used to predict the purchases of users A, B, and C. These predictions are then compared to the actual purchase records of these users. The results are shown in Table 4.

The check marks in the ‘True’ column represent the purchases made by each user, indicating their preference for the product. The red check marks in the table represent the data used to train the model. The number represents the number of reviews for the True results for a list of products, which is also the real purchase record. Because the point is to predict the products that users are interested in and recommend them to users according to the user’s real purchase records, not to predict the number of purchases, the prediction is predicted only once. The repeated purchases mean users prefer this product, which could increase the recommendation of this kind of product or similar products. In this paper, we deal with the repeated purchases as the review data. The results in Table 4 show that prediction is consistent with the true situation.

From the comparison, it can be seen that the precision of the hybrid approach prediction varies greatly from customer to customer. Observing the input and output data, it can be seen that users B and C have more purchase records than user A, and the accuracy of the recommendation results of users B and C is better than that of user A. Therefore, the more purchase records a user has, the more accurate the construction of their preference portrait, and the higher the accuracy of their recommendation.

## 4. Discussion and Conclusions

Today’s e-commerce industry is increasingly data-centric: product data and user-related data are increasingly used to provide a better service to consumers. In order to better recommend viral foods that meet user preferences to users, this paper uses reviews on the JD.com platform as experimental data.

First, Jieba word segmentation technology is used to process the review text, and a sentimental dictionary is constructed using the SO-PMI algorithm. The sentimental dictionary in the food domain provides sentimental scores for user reviews. We use the sentimental value of a food review as the user’s score for a viral food. Based on the food label attributes and user rating data, the user portraits and the food portraits are constructed.

Then, Pearson similarity and improved cosine similarity calculations are performed on the portraits. A hybrid recommendation approach is constructed that combines review text analysis and a weighted combination of the user-based collaborative filtering recommendation approach and the content-based recommendation approach.

Finally, by calculating the MAE value and accuracy, the approach is compared to the traditional recommendation approach. Our conclusions are as follows:

(1) The sentiment tendency of the review text identified using the custom domain sentiment dictionary has high accuracy, and the evaluation index of the experimental results is high. Therefore, this method is conducive to text sentiment analysis, which is an important step of the personalized recommendation system.
(2) The recommendation results of the hybrid approach vary with different parameters. It is found that, when the number of nearest neighbors, K, in the user-based collaborative filtering recommendation approach is 2, and the weight of the content-based recommendation approach is 0.9, the predictions are most accurate.
(3) By comparing the MAE value and accuracy of the hybrid approach and the traditional approaches, it is found that the content-based recommendation and the hybrid recommendation approaches are better than the user-based collaborative filtering recommendation approach. Among the content-based recommendation and the hybrid recommendation approaches, the results of the hybrid recommendation approach are slightly better.

The limited availability of user data on the platform and the difficulty of grasping the true preferences of users make improving the hybrid recommendation approach difficult. However, the viral food recommendation approach can be improved in these other aspects.

(1) Improving the domain’s sentiment words: The more comprehensive the construction of the domain’s sentiment words, the more accurate the calculation of the sentiment values. In this paper, the use of about 10,000 reviews as the basic corpus has certain limitations. The words are not comprehensive enough, resulting in low dictionary performance and sentiment score calculation performance. Therefore, it is necessary to better construct the sentimental dictionary.
(2) Improving user data information: User preference information is very important for calculating the similarity between users. It directly affects the results of the collaborative filtering recommendation approach. Owing to the protection mechanism of user privacy on the JD.com (accessed on 9 May 2021) platform, it is difficult to obtain user purchase and evaluation record data. Once the amount of review data obtained is large enough, the construction of user portraits can be more accurate.
(3) Comparing the results of multiple hybrid recommendation approaches: Different hybrid methods can be used to combine multiple approaches, and the optimal recommendation approach can be improved by continuously adjusting parameters.
(4) Validation of results from multiple aspects: Using MAE calculation and accuracy calculation methods to judge the performance of the prediction results is one-sided. The stability of the hybrid approach can be further judged based on different data sets using cross-validation methods. It would be a more comprehensive measure of the recommendation performance of the hybrid approach.

## Figures and Tables

**Table 1:** Accuracy comparisons for sentiment analysis.

	HowNet	Simplified Chinese Dictionary	Custom Dictionary
Precision	93.96%	94.45%	94.52%
Recall	92.80%	82.60%	96.40%
F1	93.38%	87.89%	95.33%

**Table 2:** Comparison of MAE of different recommendation approaches.

	CB	UserCF	Hybrid
MAE	0.131712	0.155015	0.131704

**Table 3:** Comparison of accuracy of different recommendation approaches.

	CB	UserCF	Hybrid
Precision	0.940867	0.927441	0.939712
Recall	0.893883	0.877653	0.895131
F1	0.916773	0.90186	0.91688

**Table 4:** Partial results of the empirical test.

	A		B		C	
	True	Prediction	True	Prediction	True	Prediction
Pine Cone Melaleuca Cookies Wafers	√ 1		√ 2			
Watermelon toast			√ 1		√ 1	
Barbecued Fish Tofu						
Small Twist FCL						
Mixed Candied Dried Fruit						
Waffles with Milk Flavor					√ 1	√ 1
Shredded beef jerky						
Portuguese Salted Egg Yolk Crisp		√ 1	√ 1	√ 1		√ 1
Three Squirrels Golden Meat Muffins			√ 1	√ 1		
Lactic Acid Bacteria Bread	√ 1	√ 1	√ 1			
Pig’s Trotter Marinated Flavor			√ 1		√ 1	
Snow Crisp		√ 1	√ 1	√ 1	√ 1	
Round Popping Mochi Dried Gnocchi				√ 1	√ 2	√ 1
Sandwich waffle			√ 1		√ 1	√ 1
Candy starry sky candy lollipop						
Jelly Peach Flavor						
Mimi Shrimp Crackers	√ 1	√ 1				√ 1
Biscuit pastry snack						
Cream puffs						
Tiramisu Malt Cookies	√ 2	√ 1				
Gluten roll grilled gluten		√ 1				
Spicy Beef Jerky			√ 1	√ 1		
Strawberry Marshmallow Fudge			√ 1	√ 1		√ 1
Konjac silk konjac cool						
Spicy Ezo Scallops	√ 1	√ 1				
Assorted Sandwich Cookies						
Dried Pork						
Sichuan peppercorns pepper flavor					√ 1	√ 1
Tamarind, candied fruit, dried fruit			√ 1	√ 1	√ 1	√ 1
Sandwich Seaweed			√ 2	√ 1		
Strawberry Flavor Dumpling Mochi			√ 1	√ 1	√ 1	√ 1
Seedless milk jujube and almond						
Strawberry Flavored Juice			√ 1	√ 1	√ 1	√ 1
Salted Egg Yolk Flavored Fish Skin					√ 1	√ 1
Herbal Dried Mango						√ 1
Air Chocolate Raw Chocolate	√ 1	√ 1			√ 1	√ 1
Flower cake rose cake				√ 1	√ 1	√ 1

## Data Availability

Data available on request due to privacy.
